# JAK2 Mediates the Regulation of Pept1 Expression by Leptin in the Grass Carp (*Ctenopharyngodon idella*) Intestine

**DOI:** 10.3389/fphys.2020.00079

**Published:** 2020-02-13

**Authors:** Wen-jie Luo, Peng Song, Zhi-min He, Shen-ping Cao, Jian-zhou Tang, Wen-qian Xu, Ding Xiong, Fu-fa Qu, Da-fang Zhao, Zhen Liu, Jian-zhong Li, Yu-long Yin

**Affiliations:** ^1^Hunan Provincial Key Laboratory of Nutrition and Quality Control of Aquatic Animals, Department of Biological and Environmental Engineering, Changsha University, Changsha, China; ^2^Hunan International Joint Laboratory of Animal Intestinal Ecology and Health, Hunan Normal University, Changsha, China

**Keywords:** *Ctenopharyngodon idella*, intestine, Janus kinase 2, leptin, oligopeptide transporter 1

## Abstract

Oligopeptide transporter 1 (Pept1) is located on the brush border membrane of the intestinal epithelium and plays an important role in dipeptide and tripeptide absorption from protein digestion. In this study, we cloned and characterized the cDNA sequence of Janus kinase 2 (JAK2) from *Ctenopharyngodon idella*. The expression patterns of JAK2 in various tissues and developmental stages were characterized by quantitative real-time PCR (qRT-PCR). The mRNA expression levels of JAK2 and Pept1 regulated by leptin in the intestine were also analyzed *in vitro* and *in vivo*. The cDNA sequence of JAK2 is 3378 bp in length, and the mRNA of JAK2 was broadly expressed in all tissues and embryonic stages of *C. idella* analyzed. In addition, we found that leptin regulated expression of JAK2 and Pept1 in the intestine; Pept1 expression was down-regulated by the JAK2 inhibitor AG490 *in vivo* and *in vitro*. Furthermore, luciferase experiments showed that overexpression of the JAK2 gene significantly upregulated the activity of the Pept1 5′ regulatory sequence in *C. idella*. In conclusion, these results may help in elucidating the regulatory effect of the leptin-mediated JAK2 pathway on intestinal Pept1 expression in *C. idella* and the molecular mechanism of peptide transport by the intestinal transporter Pept1 in fishes.

## Introduction

Oligopeptide transporter 1 is an oligopeptide transporter located in the brush border membrane sac of enteric epithelial cells and plays a key role in the absorption of intestinal peptides (Pan et al., 1997; Chen et al., 2002). Several studies have focused on the regulation of Pept1 transcription. For example, the transcriptional factor specificity protein 1 (Sp1) and peroxisome proliferator-activated receptor (PPAR) stimulate expression of Pept1 (Shimakura et al., 2006). In Caco2 cells, leptin regulates the Pept1 transcription via the mitogen-activated protein kinase (MAPK) pathway (Hindlet et al., 2009); leptin also enhances the activity and mRNA expression of Pept1 through the cAMP-response element-binding protein (CREB) and caudal-related homeobox 2 (CDX2) transcription factors (Nduati et al., 2007). Recent studies have demonstrated that coexpression of Janus kinase 2 (JAK2) markedly increases electronic transfer of the dipeptide glycine-glycine in *Xenopus oocytes* expressing either Pept1 or Pept2; JAK2 may also increase Pept1 activity by inserting a carrier protein into the membrane of cells, revealing that JAK2 is an influential regulator of peptide transporters (Pept1 and Pept2) (Hosseinzadeh et al., 2013).

Janus kinase is a type of non-receptor and non-transmembrane tyrosine kinase (Chen et al., 2012). JAK1, JAK2, JAK3, and TyK2 are four family members included in the JAK family (Ghoreschi et al., 2009). The molecular weights of the JAK family members range from 120 to 140 kD; the proteins consist of approximately 600 amino acids and 2 kinase regions at the N-terminus and 7 domains at the C-terminus, including 2 functional regions and 5 homologs regions (O’Shea et al., 1997). Considerable research has been performed on JAK2 in mammals (Khwaja, 2006); however, this gene has not been thoroughly characterized in fishes. According to an NCBI database search, the mRNA nucleotide sequences of JAK2 in Tetraodontidae, *Danio rerio, Cyprinus carpio, Carassius auratus*, and *Siniperca chuatsi* have been determined. The structure and tissue expression of JAK2 in fish are similar to those of the gene in mammals (Leu et al., 2000). To date, the majority of research on fish JAK2 function has concentrated on immunity. It was found that *D. rerio* JAK2a affects vascular composition and hematopoietic function (Sung et al., 2009) and also participates in interferon-γ (IFN-γ) signal transduction (Aggad et al., 2010). Furthermore, JAK2 mediates the leptin signaling pathway through substrate phosphorylation as well as in the form of a signaling complex as a scaffolding/adaptor protein (Jiang et al., 2008). Among these proteins, only the Ob-Rb isoform, which has the binding motifs required to activate the JAK/signal transducer and activator of transcription (STAT) signaling pathway, is considered to mediate the biological effects of leptin (Jiang et al., 2008). JAK2 is the kinase component in the leptin receptor signal transduction pathway (Huang and Li, 2000). The long isoform of the leptin receptor contains two binding domains for the tyrosine kinase JAK2, with one JAK2 binding domain in the short isoform (Tartaglia et al., 1995, Tartaglia, 1997). Leptin is detected in immunoprecipitates with antibodies against JAK2 but not JAK1, as tyrosine phosphorylation of JAK2 after incubation of C_2_C_12_ myotubes with leptin was observed; thus, it was assumed that leptin activates JAK2 (Kellerer et al., 1997). A previous study showed that the leptin receptor activates JAK2 kinase in hematopoietic cell lines (White, 1996). Although lacking enzymatic activity, the leptin receptor mediates intracellular signals via a connected JAK2 tyrosine kinase (Dunn et al., 2005).

Leptin is encoded by the Obese gene and belongs to a class of hormone cytokines secreted mainly by adipose cells. Obesity is a common nutritional disorder in modern society and is associated with the development and progression of non-insulin-dependent diabetes, hypertension and cardiovascular disease. Studies have shown that leptin can control animal body weight through regulating the lipid metabolism (Huang and Li, 2000). Moreover, leptin has an acute effect on the regulation of food intake, energy expenditure in fish (Li et al., 2010). Previous studies have shown that excessive JAK2 activity may contribute to development of malignancy (Venkitachalam et al., 2012). Pept1 was proved to play important roles in mediating the transportation of peptide-like drugs, such as β-lactam antibiotics and bestatin (an anticancer drug) (Inoue et al., 2005). It suggested that JAK2 and Pept1 are the key regulator or mediator in the sensitivity of tumor cells to those drugs. Our results showed that leptin regulate the expression of Pept1 via JAK2 mediated pathway, suggesting that leptin may regulate human protein metabolism-related diseases. In animals, there is a complex leptin signal transduction pathway, which exerts its biological effects mainly through the following pathways: JAK2/STAT3, phosphoinositide 3-kinase (PI3K)/protein kinase B (PKB/AKT) and MAPK/extracellular signal-regulated protein kinase 1/2 (ERK1/2) (Matarese et al., 2010). Compared with mammals, fish leptin genes are largely different from those of mammals, and research progress of the fish leptin signaling pathway also remains unclear (Kurokawa and Murashita, 2009). Currently, studies of leptin signaling pathways in fish and their biological effects are attracting widespread interest in the field of leptin research. Leptin can increase the transport efficiency of peptides across the intestinal epithelial barrier via the proton-dependent transporter Pept1 (Buyse et al., 2001). Recently, it was reported that dietary lysyl-glycine significantly increased the mRNA expression of Pept1 and leptin levels in *Oncorhynchus mykiss*, and immunohistochemistry results showed strong signals for both Pept1 and leptin on the brush border membrane of enteric epithelial cells (Ostaszewska et al., 2010). JAK2 was confirmed as one of the most critical genes in the leptin signaling pathway (Matarese et al., 2010). Pept1 is upregulated by JAK2, and peptide transport is stimulated by leptin. The pathway linking the leptin receptor to peptide transport is not known but could, at least in theory, involve JAK2 (Hosseinzadeh et al., 2013).

Therefore, in this study, we cloned and characterized the cDNA sequence of JAK2 from *Ctenopharyngodon idella*. In addition, the expression patterns of JAK2 in various tissues and developmental stages were characterized by quantitative real-time PCR (qRT-PCR). Furthermore, the mRNA expression levels of JAK2 and Pept1 regulated by leptin in the intestine were analyzed *in vitro* and *in vivo*. JAK2 inhibitor experiments and luciferase experiments also initially revealed that JAK2 mediates the regulation of intestinal Pept1 expression by leptin in *C. idella*. Our results provide valuable insight into the molecular mechanism of peptide transport in the fish intestine.

## Materials and Methods

### Animals and Sample Preparation

Healthy *C. idella* (20 g) were purchased from the Hunan Institute of Aquatic Science. All fishes were adapted to the aquaculture conditions for 1 week, and the water temperature was controlled at 24–27°C. Five *C. idella* individuals were randomly collected and anesthetized with 2-phenoxyethanol (Sigma-Aldrich, St Louis, MO, United States). Tissue samples (the hypophysis, heart, kidney, liver, spleen, muscle, and intestine) were collected for tissue distribution analysis. *C. idella* embryos were collected at different developmental stages (fertilized egg, gastrula stage, neurologic stage, organ stage, hatching stage, 1 d post-hatch, 4 d post-hatch, and 7 d post-hatch) and stored at −80°C until further analysis of JAK2 mRNA expression.

### RNA Isolation and cDNA Synthesis

Total RNA was isolated from the tissues as indicated using TRIzol reagent (Invitrogen, Carlsbad, CA, United States). The quality and concentration of total RNA was detected through agarose gel electrophoresis and a nucleic acid protein analyzer (Eppendorf, Germany) based on the A260/A280 ratio. Subsequently, 1 μg of total RNA was reverse transcribed to cDNA with oligo (dT) primers and a cDNA Synthesis Kit (TaKaRa, Japan) according to the manufacturer’s instructions.

### JAK2 cDNA Cloning and Sequence Identification

The full-length nucleotide sequence of JAK2 from different animals was downloaded from NCBI (GenBank: MK330872.1), and degenerate primers for conserved regions were designed using Primer Premier 5.0 (Table 1). PCR amplifications were performed as follows: an initial denaturation at 95°C for 5 min followed by 34 cycles of denaturation at 95°C for 30 s, annealing at 57°C for 30 s and extension at 72°C for 1 min followed by a final extension at 72°C for 10 min. The PCR products were evaluated by 1.5% agarose gel electrophoresis; the products were double-digested with restriction enzymes, ligated to the vector pMD19-T (Takara, Japan) and sequenced using an Applied Biosystems (ABI) 3730 DNA Sequencer. The nucleotide sequences of JAK2 were analyzed using the BLAST network service at NCBI^1^. The amino acid sequence and protein structure were characterized using the translation tool software ExPASy^2^. The identity and similarity of the amino acid sequence were examined using MatGAT2.02 software (Nassl et al., 2011). Sequence alignments were performed to compare the amino acid sequences of JAK2 with other vertebrate homologs using the MegAlign program with the Clustal W method. Phylogenetic trees were generated through a neighbor-joining (NJ) method with MEGA 5.0 (Hart et al., 2016). The confidence of each node was assessed by 1000 bootstrap replicates.

**TABLE 1:** Sequences of designed primers used in this study.

Primer	Sequence (5′–3′)	Comment
JAK2-F	TTGACCCCAAACATCTTAGCG	CDS Cloning
JAK2-R	TCAAGTCCTGGGGTTAGAT	
JAK2-Flag- *Bam*HI-F	CGCGGATCCATGGAGACAGCTGTCTGT	pCMV-N-Flag-JAK2
JAK2-Flag-*Xho*I-R	CCGCTCGAGTTAAAATGCAGGCACTTG	
PepT1-pGL3- F	CGGGGTACCGCAGTTAGGCGATGA	PGL3-Basic- PepT1
PepT1-pGL3- R	CCGCTCGAGCTCTGAGGGAGACAG	
PepT1-R1	TGCCATAGTAGGAGAATCGC	PepT1 Promoter
PepT1-R2	CTCTGAGGGAGACAGGTAACAAGG	
PepT1-R3	CTCTGAGGGAGACAGGTAACAAGG	Real-Time PCR
JAK2-qPCR-F	GAGAATGGCGACTACAATC	
JAK2-qPCR-R	GAACAGCACCTGCTAAACTG	
PepT1-qPCR-F	TGCTCTTGTTGTGTTCATCG	Real-Time PCR
PepT1-qPCR-R	CTCTCTCTTGGGGTATTGCTT	
β-actin-F	CCTTCTTGGGTATGGAGTCTTG	Real-Time PCR
β-actin-R	AGAGTATTTACGCTCAGGTGGG	

### Quantitative Real-Time PCR

The mRNA expression levels of JAK2 were analyzed by qRT-PCR with QuantStudio^®^ 3 Real-Time PCR (Applied Biosystems). The gene-specific primers used for qRT-PCR are listed in Table 1. *C. Idella*β-actin was used as an internal control to normalize the samples. Real-time PCR was performed in triplicate for each complementary DNA sample using SYBR Green I in a final volume of 12.5 μL (6 μL of SYBR^®^ Premix Ex Taq (2×), 0.5 μL cDNA, 0.5 μL of each primer (10 μM) and 5 μL of ddH_2_O). The PCR conditions were as follows: 95°C for 30 min followed by 40 cycles at 95°C for 10 s and 60°C for 30 s. To maintain consistency, the baseline was set automatically by the software. The relative mRNA expression level was analyzed using the comparative CT method $\left[2^{-\Delta \Delta \text{Ct}\;}\left(\Delta \Delta {\text{C}}_{\text{t}}={\left({\text{C}}_{\text{t}\;\text{gene}\;\;\text{of}\;\text{interest}}-{\text{C}}_{\text{t}\;\;\text{β-actin}}\right)}_{\text{treat}}-\;{\left({\text{C}}_{\text{t}\;\text{gene}\;\;\text{of}\;\text{interest}}-{\text{C}}_{\text{t}\;\;\text{β-actin}}\right)}_{\text{untreat}}\right)\right]\;$ with CFX Manager software (Chaoyue et al., 2019).

### Intraperitoneal Injection and *in vitro* Leptin Experiments

*Ctenopharyngodon idella* individuals (20 g; *n* = 72) after 1 week of domestication were selected for intraperitoneal injection of leptin at different concentrations. Research has shown that, grass carp weighing 14.26 ± 0.22 g was injected with 30 μg LEP (Li et al., 2010). According to the weight of our experimental *C. idella*, we conducted our study at the concentration gradient of leptin addition. The experiment was performed by injecting 200 μL of phosphate-buffered saline (PBS) as the control group and injecting 200 μL leptin at doses of 0.016 μg/g, 0.16 μg/g, 1.6 μg/g, and 16 μg/g into the experimental group. The protein concentration was measured using non-interference-type protein quantification kit (Cat. No.:C503071). The intraperitoneal injection experiments were designed with 3 fish repeats and set to 4 repetitions per group. The experiment was carried out in the indoor circulation culture system of the Aquaculture Base of the College of Biotechnology, Changsha University. At 8 h after intraperitoneal injection, samples were collected to detect relative JAK2 and Pept1 mRNA expression by RT-PCR.

For *in vitro* experiments, *C. idella* individuals (20 g) were anesthetized with 2-phenoxyethanol, and their intestines were rapidly separated using scissors. After three washes with PBS, the intestines were incubated with 0.05% (weight/volume) collagenase (Sigma-Aldrich, St. Louis, MO, United States) in PBS for 15 min, followed by three additional washes with PBS and centrifugal collection of cells. Intestinal primary cells were cultured in 24-well culture plates with 1 mL Dulbecco’s modified Eagle’s medium (DMEM) containing 10% fetal bovine serum (FBS, Gibco BRL, United States) per well at 28°C with 5% CO_2_ for 1 week; the medium was changed every 2 days during this period. After subculture, the second-generation *C. idella* intestinal cells were inoculated into 6-well plates and randomly divided into 6 groups of 4 wells. After passaging, the cells had adhered well and were normally extended (4th day). The original culture solution was aspirated using a sterile pipette and gently washed with 2 mL PBS. Complete cell medium containing different concentrations of *C. idella* leptin (0, 50, 100, 250 and 500 ng/mL) was added to each group of cells. After 8 h of culture, the cell culture medium was removed, the cells were collected with a scraper and suspended with PBS; the cells were collected by centrifugation, and the relative mRNA expression levels of JAK2 and Pept1 were detected by RT-PCR.

### Inhibitor Experiments *in vivo* and *in vitro*

*Ctenopharyngodon idella* fishes were selected (*n* = 48) and randomly divided into four groups for the JAK2-specific inhibitor AG490 injection experiment. Four groups of *C. idella* were injected with PBS, leptin, leptin + AG490, and AG490, and 200 μL PBS was injected as the control group, three repeats per group. At 8 h after injection, samples (intestine) were collected, and relative expression of Pept1 mRNA was detected by RT-PCR.

For *in vitro* experiments, *C. idella* intestinal cells in 16-well plates were randomly divided into four groups. No other substances were added to the complete culture medium of the control group. Leptin, leptin + AG490 and AG490 were added to the complete culture medium of the three experimental groups, with three repeats per group. After 8 h of culture, the cells were collected, and relative mRNA expression of Pept1 was detected by RT-PCR.

### Preparation of Reporter Constructs

*Ctenopharyngodon idella* enteric DNA was extracted using TaKaRa MiniBEST Universal Genomic DNA Extraction Kit Ver. 5.0; the Pept1 gene promoter was cloned using Genome Walking Kit, and the PCR product was purified using TaKaRa MiniBEST DNA Fragment Purification Kit Ver. 4.0. The promoter of the Pept1 gene was cloned using Genome Walking Kit. The pCMV-N-Flag and PGL3-basic (Promega) vectors were digested with *Bam*HI and *Xho*I and purified using a plasmid purification kit (Magen); JAK2 from *C. idella* and the promoter of the Pept1 gene with *Bam*HI and *Xho*I double restriction sites were purified. The fragments were ligated overnight and transformed into competent *DH5*α cells. Positive clones were selected. After successful sequencing, cultures were expanded, and the pCMV-N-Flag-JAK2 and pGL3-basic-Pept1 recombinant plasmids were purified using a high-copy low endotoxin plasmid extraction kit (HiPure Plasmid EF Midi Kit, Magen).

### Dual-Luciferase Reporter Assay

HEK293T cells were cultured in 10-mm culture plates with 10 mL of DMEM containing 10% FBS (Gibco BRL, United States) per well at 37°C with 5% CO_2_ for 1 week. Prior to transfection, the culture medium was removed, and the HEK293T cells adhered to the plate. The cells were transfected in serum-free DMEM using Lipofectamine 2000 (Invitrogen) at a 1:2 ratio of DNA (pCMV-N-Flag-JAK2/pGL3-basic-Pept1) to transfection reagent. At 6 h post-transfection, the medium was removed from the 6-well plate, and complete medium containing 10% FBS and antibiotics was added; the culture was continued for 1 day, after which the cell solution was transferred to a 1.5-mL centrifuge tube, followed by centrifugation at 3000 rpm for 5 min at 4°C. The supernatant was removed, and the cells were stored at −80°C.

Luciferase reporter gene analysis was performed using Dual-Luciferase Reporter Assay System (Promega) kit. HEK293T cells were transfected with plasmids containing the luciferase gene and the relevant Pept1 promoter. After stimulation, the resulting luminescence was measured for 10 s with a luminometer. Extracts were analyzed in triplicate, and each experiment was performed three times.

### Statistical Analysis

The statistical analysis was performed with SPSS 18.0 software. One-way analysis of variance (ANOVA) was employed to determine differences among groups. All data are expressed as the mean ± SE in each of the independent experiments. *P* < 0.05 was considered to indicate statistical significance.

## Results

### Isolation and Sequence Analysis of JAK2 in *C. idella*

**FIGURE 1:**
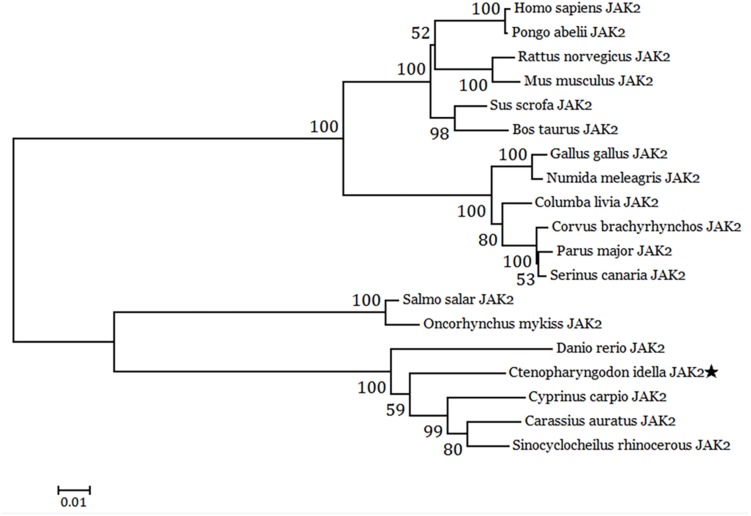
Neighbor-joining phylogenetic tree of JAK2 from 19 vertebrate species (*C. idella* JAK2 is asterisked).

**FIGURE 2:**
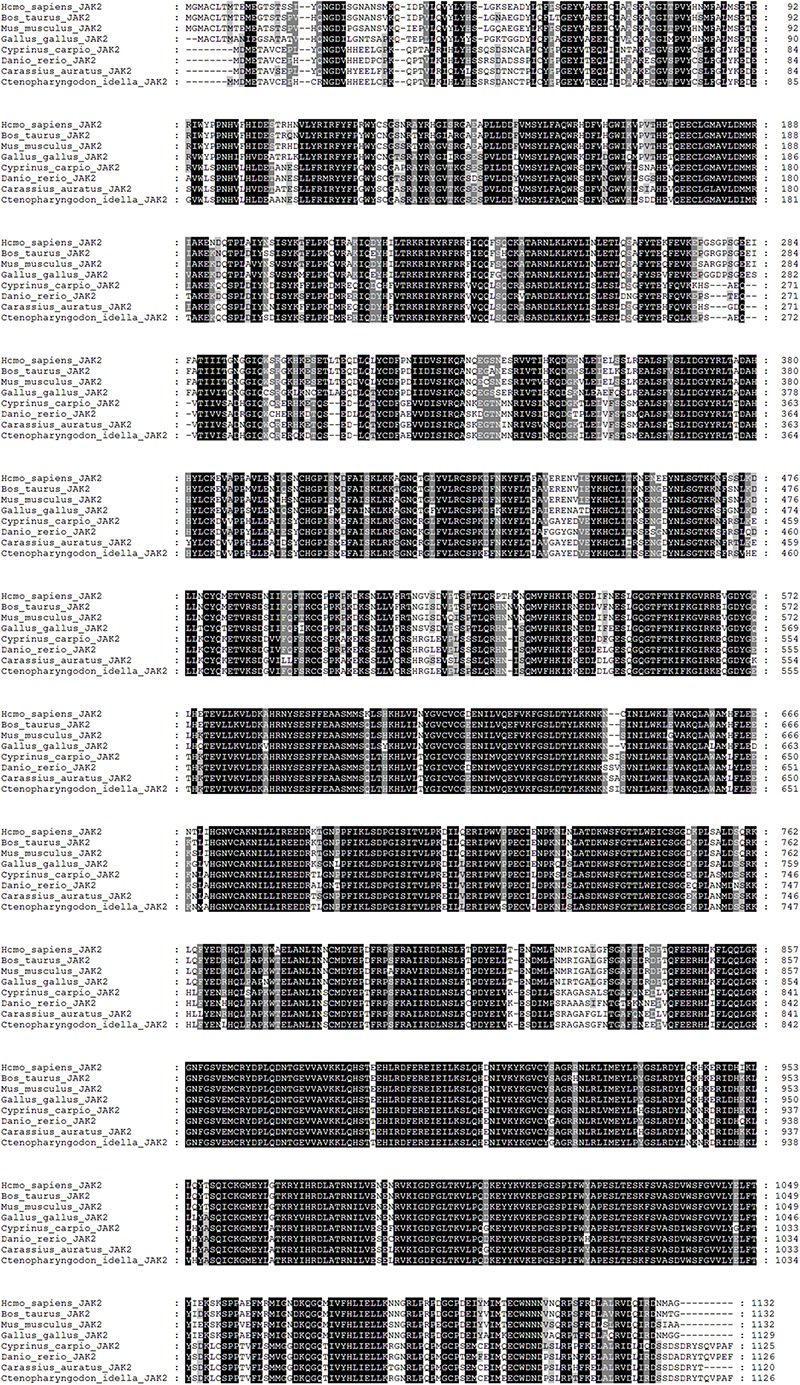
Alignment analysis of the deduced amino acid sequence in JAK2.

The cDNA sequence of JAK2 (No. MK330872) was isolated from an intestine cDNA library for *C. idella.* Subsequently, the characteristics of JAK2 were analyzed. The results showed that JAK2 is encoded by a 3378-bp gene. The molecular weight of the encoded protein is 12950.26 Da, and its theoretical pI is 6.37. The results of phylogenetic analysis showed that *C. idella* JAK2 is subordinate to the branch of fish, which is consistent with traditional taxonomy (Figure 1). The JAK2 amino acid sequence shows high similarity, from 96.2 to 97.2%, with those of *D. rerio*, *C. carpio*, *C. auratus*, and *I. rhinocerous*, as well as significant identity from 91.5 to 94.0% (Table 2). The conserved amino acid sequences of JAK2 among *C. idella*, *Homo sapiens*, *Bos taurus*, *Mus musculus*, *Gallus gallus*, *C. carpio*, *C. auratus*, and *D. rerio* are marked with dark shadows in Figure 2.

**TABLE 2:** Comparison of amino acids between grass carp JAK2 and other known species JAK2.

Species	GenBank access. no.	Similarity	Identity
*Homo sapiens*	NP_001309125.1	84.6	68.8
*Sus scrofa*	BAA21662.1	84.8	69.7
*Bos taurus*	XP_005210038.1	84.7	69.4
*Rattus norvegicus*	NP_113702.1	84.8	69.3
*Pongo abelii*	NP_001125600.1	84.6	68.9
*Mus musculus*	NP_032439.2	84.8	69.4
*Equus caballus*	XP_001492713.1	85.0	69.5
*Gallus gallus*	NP_001025709.2	83.1	69.2
*Numida meleagris*	XP_021235768.1	79.8	67.6
*Columba livia*	XP_013227099.1	82.6	69.2
*Parus major*	XP_015508092.1	83.2	68.6
*Serinus canaria*	XP_009097237.1	83.0	68.7
*Corvus brachyrhynchos*	XP_017588292.1	83.5	68.8
*Cyprinus carpio*	AJP77390.1	97.2	92.9
*Danio rerio*	NP_571162.1	96.7	91.5
*Carassius auratus*	XP_026067978.1	96.2	91.8
*Salmo salar*	XP_014027024.1	89.5	77.4
*Oncorhynchus mykiss*	XP_021461243.1	89.5	77.2
*Sinocyclocheilus rhinocerous*	XP_016383392.1	97.1	94.0

### Expression Pattern of JAK2 in Various Tissues and Embryonic Stages

**FIGURE 3:**
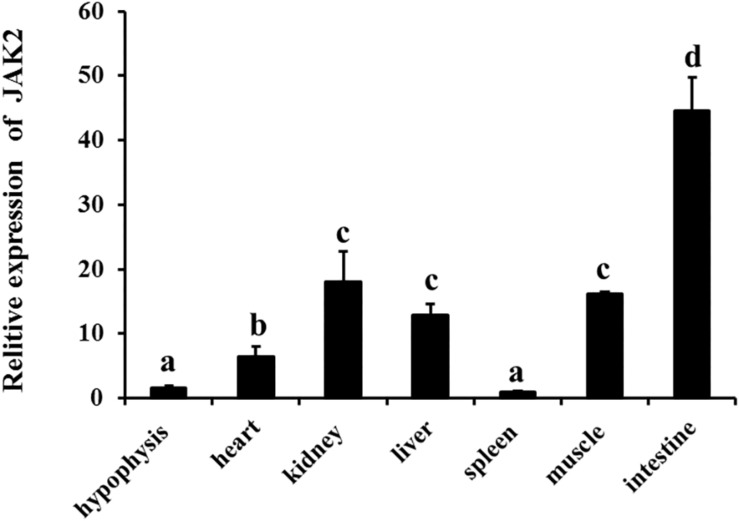
Relative expression level of JAK2 mRNA in tissues of *C. idella.* Values in rows with different letters are considered as significantly different (*P* < 0.05).

qRT-PCR was employed to analyze JAK2 mRNA expression. The results showed that JAK2 of *C. idella* was broadly expressed in all selected tissues, including the hypophysis, heart, kidney, liver, spleen, muscle, and intestine. The level of JAK2 gene expression was highest in the intestine, with a significant difference compared with other tissues (*P* < 0.05), and followed by the kidney, muscle, heart, and liver; expression was lowest in the spleen. In addition, the expression levels of the JAK2 gene in the heart, kidney, liver, and muscle were significantly higher than those in the hypophysis and spleen (*P* < 0.05) (Figure 3).

**FIGURE 4:**
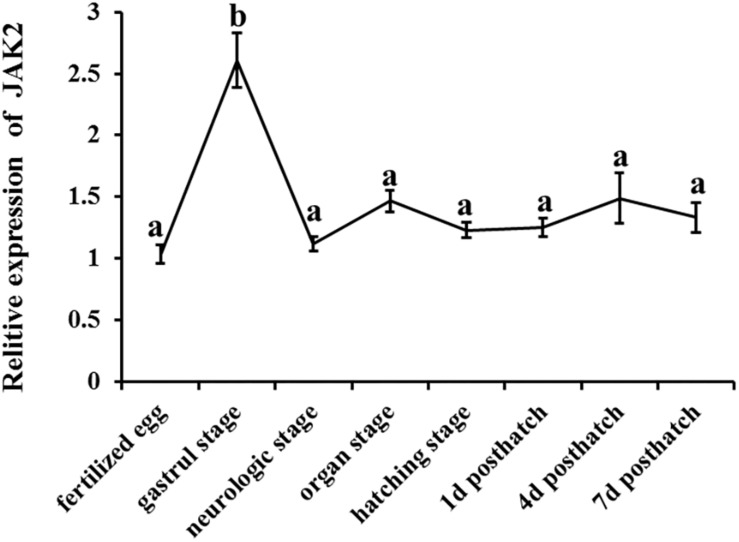
The tendency of Relative mRNA expression level of JAK2 in embryogenesis developmental stages of *C. idella*. Values in rows with different letters are considered as significantly different (*P* < 0.05).

The expression patterns of JAK2 were also evaluated in different embryonic stages of *C. Idella*, including fertilized eggs, gastrula stage, neurological stage, organ stage, hatching stage, 1 d post-hatch, 4 d post-hatch, and 7 d post-hatch. According to the results, JAK2 mRNA was expressed in all the selected stages of embryonic development, with dynamic changes; the expression level in the gastrula stage was dramatically higher than that in the other periods (*P* < 0.05); expression in the other seven periods was largely stable, with no significant difference (*P* > 0.05) (Figure 4).

### Effects of Leptin on Intestinal JAK2 and Pept1 Expression *in vivo* and *in vitro*

**FIGURE 5:**
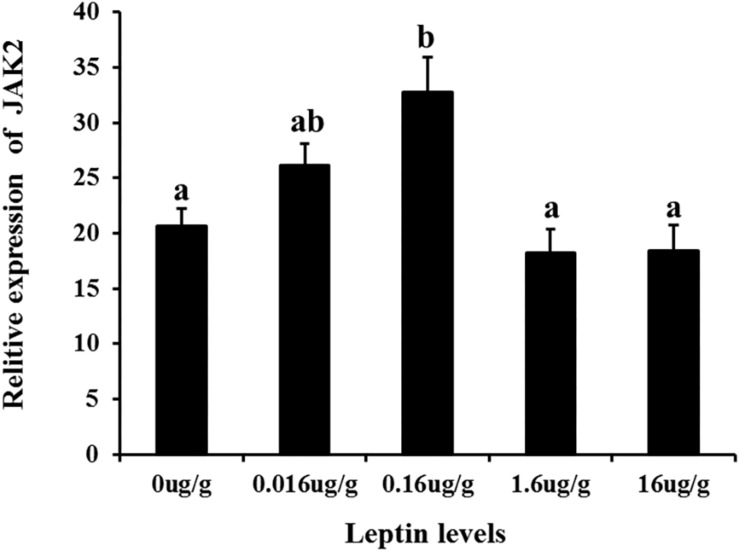
The effects of leptin injection using different levels of on the relative mRNA expression of JAK2 in intestine of *C. idella*. Values in rows with different letters are considered as significantly different (*P* < 0.05).

**FIGURE 6:**
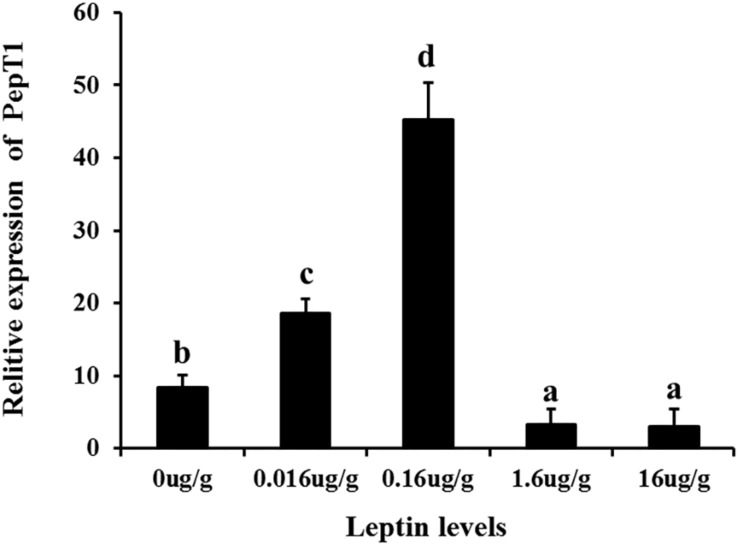
The Effects of leptin injection using different levels of on the relative mRNA expression of PepT1 in intestine of *C. idella*. Values in rows with different letters are considered as significantly different (*P* < 0.05).

To investigate the effects of leptin on *C. idella*, the mRNA expression levels of JAK2 and Pept1 were examined by qRT-PCR *in vivo* and *in vitro* after treating *C. idella* with different concentrations of leptin. The expression level of the JAK2 gene was highest in fishes treated with 0.16 μg/g leptin (*P* < 0.05), and there was no significant difference among the other groups (*P* > 0.05) (Figure 5). Moreover, expression of the Pept1 gene in the intestine of *C. idella* first increased and then decreased with increasing leptin concentration, reaching the peak level at 0.16 μg/g, which was significantly higher than that in the other five groups (*P* < 0.05) (Figure 6). The expression levels of the Pept1 gene in the 1.6 and 16 μg/g leptin groups were lower than those in the first three groups (*P* < 0.05). Furthermore, the expression levels of the JAK2 and Pept1 genes in the intestine of *C. idella* displayed a tendency to decline at the beginning and rise in late stages, again with the highest level at 0.16 μg/g. Our findings suggest that JAK2 and Pept1 mRNA expression trends were consistent under different concentrations of leptin treatment.

**FIGURE 7:**
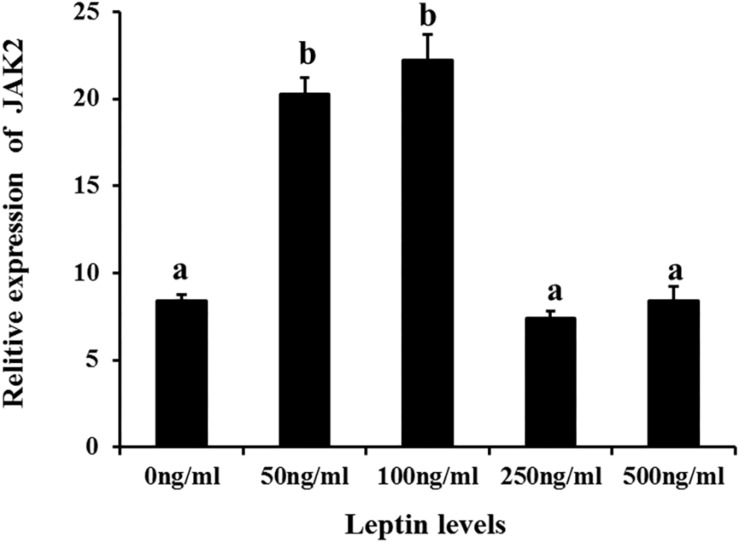
Effects of different levels of leptin on the relative expression of JAK2 in intestinal cells of *C. idella*. Values in rows with different letters are considered as significantly different (*P* < 0.05).

**FIGURE 8:**
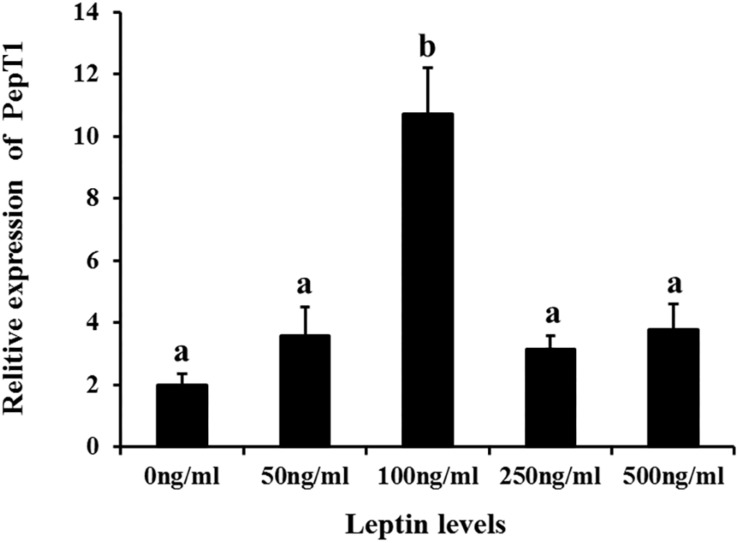
Effects of different levels of leptin on the relative expression of PepT1 in intestinal cells of *C. idella*. Values in rows with different letters are considered as significantly different (*P* < 0.05).

Additionally, to determine changes in JAK2 and Pept1 mRNA expression, similar leptin treatments were applied to *C. idella* intestinal cells. JAK2 gene expression was high in the 50 and 100 ng/mL leptin groups (*P* < 0.05) (Figure 7). Pept1 mRNA expression reached a peak in the 100 ng/mL leptin group (*P* < 0.05) (Figure 8), though there was no significant difference among the other groups (*P* > 0.05).

### Effects of AG490 on Intestinal Pept1 Expression *in vivo* and *in vitro*

**FIGURE 9:**
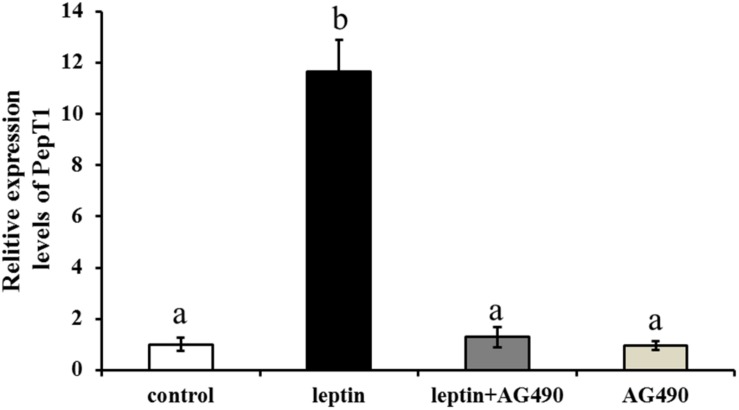
Effects of leptin and AG490 injection on the relative expression of PepT1 mRNA in intestinal tissues of *C. idella*. Values in rows with different letters are considered as significantly different (*P* < 0.05).

To study the effects of the specific JAK2 inhibitor AG490 and leptin on the expression of Pept1, AG490 and leptin were intraperitoneally injected into *C. idella.* JAK2 activity was clearly affected by AG490. After 8 h of treatment, the level of Pept1 gene expression in the intestine of *C. idella* peaked in the group injected with 0.16 μg/g leptin (*P* < 0.05), with no significant difference among the other three groups (*P* > 0.05) (Figure 9).

**FIGURE 10:**
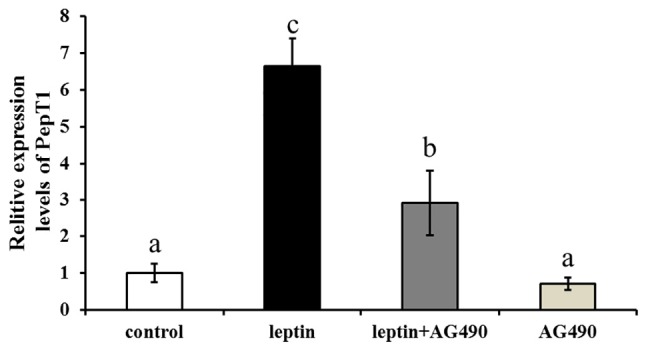
Effects of leptin and AG490 injection on the relative expression of PepT1 mRNA in intestinal cells of *C. idella*. Values in rows with different letters are considered as significantly different (*P* < 0.05).

*Ctenopharyngodon idella* intestinal cells were treated with the JAK2-specific inhibitor AG490 and with 100 ng/mL leptin for 8 h, followed by qRT-PCR to detect Pept1 expression. The expression level of the Pept1 gene reached a peak in the medium supplemented with leptin alone (*P* < 0.05) followed by leptin with AG490 (*P* < 0.05) and was lowest in the control and AG490 groups (*P* < 0.05) (Figure 10).

### Dual-Luciferase Reporter Assay

**FIGURE 11:**
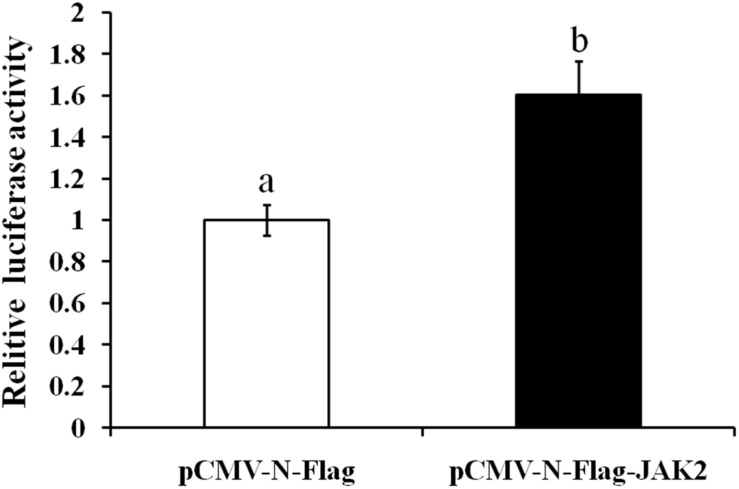
Effects of overexpression of JAK2 on the activity of the PepT1 5′ terminal regulatory sequence in HEK293T cells. Values in rows with different letters are considered as significantly differents (*P* < 0.05).

In this assay, HEK293T cells were used as model cells for performing a luciferase assay to detect regulation of Pept1 gene activity by pCMV-N-Flag-JAK2. The pCMV-N-Flag vector was used as a blank control, and pRL-TK served as an internal reference. Four replicates were applied for each group. The results showed that overexpression of the JAK2 gene significantly upregulated the promoter activity of the Pept1 sequence (*P* < 0.05) (Figure 11).

## Discussion

Studies have shown that the JAK2 amino acid sequence of close species has high similarity (Leu et al., 2000). The amino acid encoded by the human JAK2 gene is similarity to that of other mammals, reaching 95% (Saltzman et al., 1998); the similarity of the JAK2 amino acid sequence between *Takifugu rubripes* and *S. chuatsi* is 89%, and that between *T. rubripes* and rats is 66.8% (Guo et al., 2009). In this study, the cDNA sequence of the *C. idella* JAK2 gene obtained by homologous cloning technology is 3676 bp long, with an ORF of 3378 bp that encodes 1126 amino acids. Phylogenetic analysis shows that the JAK2 amino acids of 19 selected species can be clustered into three branches, including mammals, birds and fishes. High consistency with the *C. idella* JAK2 sequence was found for *D. rerio*, *C. carpio*, *C. auratus*, and *I. rhinocerous*, all above 91%, though low consistency was observed for *Salmo salar* (77.4%) and *O. mykiss* (77.2%), which may be attributable to their different families: Cyprinidae and Salmonidae. Amino acid sequence alignment showed that the JAK2 gene is well conserved among species. The 3396-bp ORF (containing a stop codon) of the human JAK2 protein encodes 1132 amino acids (Saltzman et al., 1998). *C. idella* JAK2 is 6 amino acids shorter than human JAK2. This difference may be related to the functional diversity of JAK2 during evolution and variability in species.

Studies in a many animals have shown that JAK2 is distributed in various tissues of the organisms. For example, northern blot hybridization revealed human JAK2 to be broadly expressed in the placenta, liver, thymus, skeletal muscle, kidney, lung, spleen, testis, ovary, pancreas, heart, brain, small intestine, colon, and peripheral haemolymph (Saltzman et al., 1998). Similarly, rat JAK2 was also found to be expressed in various tissues, mainly in the brain and spleen and to a lesser extent in the heart and kidney (Neubauer et al., 1997). Previous studies have shown that in fishes, the distribution of JAK2 gene expression has the same characteristics of wide distribution in tissues but with variability among species. *S. chuatsi* JAK2 is expressed in the sputum, spleen, heart, brain, intestine, kidney, gonad, and liver, especially in gonads (Guo et al., 2009). In this study, the JAK2 gene was quantitatively detected by qRT-PCR in several tissues of *C. Idella*, being expressed in all tissues tested, with especially high levels in the intestine and low levels in the hypophysis and spleen.

Janus family kinases (JAKs) are non-receptor protein tyrosine kinases that are involved in the growth, survival, development and differentiation of a variety of cells and play an important role in immune and hematopoiesis (Duhé et al., 1995; Dalal et al., 1998; Saltzman et al., 1998; Radosevic et al., 2004; Frenzel et al., 2006). The gastrula is an important stage in the development of animal embryos that derive from a blastula and have double or triploblastic embryos (Liu et al., 2005). In the gastrula stage, the nucleus plays a major role and lays the foundation for synthesizing new proteins and cell differentiation for tissue and organogenesis (Liu et al., 2005). In this study, the highest expression level of *C. idella* JAK2 in the gastrula stage suggests that it might participate in cell differentiation of the gastrointestine. Simultaneously, JAK2 expression varies with the individual development stage of species. Some studies have shown that erythrocytosis is mediated by JAK2a activation *D. rerio* embryos. The JAK2a gene is expressed at high levels in erythroid precursors of primitive and definitive waves and at a lower level in the early central nervous system and developing fin buds, and it may be functionally equivalent to mammalian JAK2 in early erythropoiesis (Sung et al., 2009).

In fishes, research on leptin has mainly focused on feeding and lipid metabolism. Injection of leptin resulted in a strong anorexia response in *O. mykiss*, which was consistent with the transient decrease of the mRNA level of appetitive neuropeptide Y (NPY) and enhanced mRNA levels of anorexia melanotropic corticotropin (ACTH) A1 and A2 (Murashita et al., 2008). Intraperitoneal injection of leptin inhibited expression of lipase metabolism-related genes in *C. idella.* Leptin also promotes expression of the genes encoding pancrelipase and fatty acid-lengthening enzyme (Li et al., 2010). JAK2 mediates leptin signaling by both phosphorylating its substrates and forming a signaling complex as a scaffolding/adaptor protein, and gastric leptin might control the absorption of dietary proteins by modulating the activity of Pept1 (Buyse et al., 2001; Jiang et al., 2008). In this study, different concentrations of leptin were injected intraperitoneally *in vivo*, and a low concentration significantly induced expression of JAK2 and Pept1 in the intestine of *C. idella* after 8 h. This result revealed that leptin is involved in the regulation of intestinal JAK2 and Pept1 expression in *C. idella*. JAK2 increases Pept1 activity by promoting its expression on the cell membrane, and a recent study reported that the pharmacological JAK2 inhibitor AG490 decreases the peptide-induced current in segments of mouse small intestine, an observation highlighting the *in vivo* significance of JAK2-mediated regulation of peptide transporters (Hosseinzadeh et al., 2013). The effect of JAK2 was reversed by AG490, and our inhibitor experiments showed that JAK2 possibly mediated regulation of Pept1 by leptin. Importantly, our results verified a previous hypothesis that the signaling pathway linking the leptin receptor to peptide transport involves JAK2. Leptin regulates peptide transport and absorption via the leptin-JAK2-Pept1 pathway, suggesting that the JAK2 gene is involved in the regulation of Pept1 in the intestine of *C. idella* and indirectly affects the transport of small peptides via Pept1. As the selectivity of pharmacological inhibitors may be limited, it remains uncertain whether JAK2 indeed participates in the regulation of intestinal peptide transport. These results indicate that JAK2 may regulate Pept1.

In mammals, the JAK2-STAT signaling pathway plays a key role in leptin signaling. A few studies have suggested that leptin influences Pept1 translocation in the rat jejunum and human intestinal Caco-2 cells. Pept1 and leptin receptors are reportedly expressed in Caco-2 and rat intestinal mucosal cells (Buyse et al., 2001; Matarese et al., 2010). Our research showed that leptin can regulate expression of JAK2 and Pept1 and that and expression of Pept1 interfered with the JAK2 inhibitor AG490 *in vivo* and *in vitro*. However, whether leptin can regulate Pept1-mediated transport of small peptides through the JAK2 signaling pathway in the *C. idella* intestine remains unclear. In this study, a recombinant plasmid expressing *C. idella* JAK2 was constructed. The dual-luciferase reporter assay showed that JAK2 gene overexpression significantly upregulated the promoter activity of the 5′ terminal regulatory sequence of Pept1, suggesting that JAK2 in *C. idella* intestine may have a regulatory effect on Pept1 expression.

In summary, our study preliminarily reveals the regulatory mechanism of leptin on Pept1 via the JAK2 pathway at mRNA level and helps to elucidate the mechanism of transport and absorption of oligopeptides. We have tried to use human JAK2 antibody [anti JAK2 (py1007) antibody, fusion Biosciences] to analyze the effect of leptin on JAK2 protein level, but because the JAK2 antibody has low specificity, it can not act on grass carp. Therefore, it is necessary to further prepare grass carp JAK2 antibody to study the effect of leptin protein on JAK2 protein level. JAK2 is not directly acting on Pept1 and which master regulator or transcription factor driving the effect of Pept1 on intestine still need to identify. Besides, further studies are needed to elucidate the leptin-mediated signal transduction pathway between JAK2 and Pept1 in the intestine of *C. idella*.

## Data Availability Statement

The raw data supporting the conclusions of this article will be made available by the authors, without undue reservation, to any qualified researcher.

## Ethics Statement

All procedures complied with the ARRIVE guidelines and were carried out in accordance with the UK Animals (Scientific Procedures) Act of 1986 and associated guidelines. The animal study was reviewed and approved by the committee on animal care of the Changsha University and under protocol no. CU-2018-10-24-003.

## Author Contributions

WL, PS, ZH, SC, JT, WX, DX, FQ, and DZ conducted the experiments. WL, ZL, and JL collected and analyzed the data. ZL, JL, and YY helped with the discussion. WL, ZL, and JL designed the experiments and revised the manuscript.

## Conflict of Interest

The authors declare that the research was conducted in the absence of any commercial or financial relationships that could be construed as a potential conflict of interest.

## Footnotes

1 http://blast.ncbi.nlm.nih.gov/

2 http://www.expasy.org/tools/

## References

[B1] Aggad D. Stein C. Sieger D. Mazel M. Boudinot P. Herbomel P. (2010). *In vivo* analysis of Ifn-gamma1 and Ifn-gamma2 signaling in zebrafish. *J. Immunol.* 185 6774–6782. 10.4049/jimmunol.1000549 21048110

[B2] Buyse M. Berlioz F. Guilmeau S. Tsocas A. Voisin T. Peranzi G. (2001). PepT1-mediated epithelial transport of dipeptides and cephalexin is enhanced by luminal leptin in the small intestine. *J. Clin. Invest.* 108 1483–1494. 10.1172/JCI13219 11714740 PMC209419

[B3] Chaoyue W. Fengna L. Yehui D. Qiuping G. Wenlong W. Lingyu Z. (2019). Dietary taurine regulates free amino acid profiles and taurine metabolism in piglets with diquat-induced oxidative stress. *J. Funct. Foods* 62:103569 10.1016/j.jff.2019.103569

[B4] Chen E. Staudt L. M. Green A. R. (2012). Janus kinase deregulation in leukemia and lymphoma. *Immunity* 36 529–541. 10.1016/j.immuni.2012.03.017 22520846 PMC7480953

[B5] Chen H. Pan Y. Wong E. A. Bloomquist J. R. Webb K. E. Jr. (2002). Molecular cloning and functional expression of a chicken intestinal peptide transporter (cPepT1) in *Xenopus oocytes* and Chinese hamster ovary cells. *J. Nutr.* 132 387–393. 10.1093/jn/132.3.387 11880560

[B6] Dalal I. Enrico A. Harjit D. Shaila K. Jerami S. Chaim M. R. (1998). Cloning and characterization of the human homolog of mouse Jak2. *Blood* 91 844–851. 10.1182/blood.V91.3.844.844_844_851 9446644

[B7] Duhé R. J. Rui H. Greenwood J. D. Garvey K. Farrar W. L. (1995). Cloning of the gene encoding rat JAK2, a protein tyrosine kinase. *Gene* 158 281–285. 10.1016/0378-1119(95)00041-4 7607555

[B8] Dunn S. L. Bjornholm M. Bates S. H. Chen Z. Seifert M. Myers M. G. Jr. (2005). Feedback inhibition of leptin receptor/Jak2 signaling via Tyr1138 of the leptin receptor and suppressor of cytokine signaling 3. *Mol. Endocrinol.* 19 925–938. 10.1210/me.2004-0353 15604114

[B9] Frenzel K. Wallace T. A. McDoom I. Xiao H. D. Capecchi M. R. Bernstein K. E. (2006). A functional Jak2 tyrosine kinase domain is essential for mouse development. *Exp. Cell Res.* 312 2735–2744. 10.1016/j.yexcr.2006.05.004 16887119

[B10] Ghoreschi K. Laurence A. O’Shea J. J. (2009). Janus kinases in immune cell signaling. *Immunol. Rev.* 228 273–287. 10.1111/j.1600-065X.2008.00754.x 19290934 PMC2782696

[B11] Guo C. Zhang Y. Yang L. Yang X. Wu Y. Liu D. (2009). The JAK and STAT family members of the mandarin fish *Siniperca chuatsi*: molecular cloning, tissues distribution and immunobiological activity. *Fish Shellfish Immunol.* 27 349–359. 10.1016/j.fsi.2009.06.001 19539032

[B12] Hart H. R. Evans A. N. Gelsleichter J. Ahearn G. A. (2016). Molecular identification and functional characteristics of peptide transporters in the bonnethead shark (*Sphyrna tiburo*). *J. Comp. Physiol. B* 186 855–866. 10.1007/s00360-016-0999-8 27188191

[B13] Hindlet P. Bado A. Kamenicky P. Delomenie C. Bourasset F. Nazaret C. (2009). Reduced intestinal absorption of dipeptides via PepT1 in mice with diet-induced obesity is associated with leptin receptor down-regulation. *J. Biol. Chem.* 284 6801–6808. 10.1074/jbc.M805564200 19144638 PMC2652284

[B14] Hosseinzadeh Z. Dong L. Bhavsar S. K. Warsi J. Almilaji A. Lang F. (2013). Upregulation of peptide transporters PEPT1 and PEPT2 by Janus kinase JAK2. *Cell. Physiol. Biochem.* 31 673–682. 10.1159/000350086 23711493

[B15] Huang L. Li C. (2000). Leptin: a multifunctional hormone. *Cell Res.* 10 81–92. 10.1038/sj.cr.7290038 10896170

[B16] Inoue M. Terada T. Okuda M. Inui K. (2005). Regulation of human peptide transporter 1 (PEPT1) in gastric cancer cells by anticancer drugs. *Cancer Lett.* 230 72–80. 10.1016/j.canlet.2004.12.023 16253763

[B17] Jiang L. Li Z. Rui L. (2008). Leptin stimulates both JAK2-dependent and JAK2-independent signaling pathways. *J. Biol. Chem.* 283 28066–28073. 10.1074/jbc.M805545200 18718905 PMC2568905

[B18] Kellerer M. Koch M. Metzinger E. Mushack J. Capp E. Haring H. U. (1997). Leptin activates PI-3 kinase in C2C12 myotubes via janus kinase-2 (JAK-2) and insulin receptor substrate-2 (IRS-2) dependent pathways. *Diabetologia* 40 1358–1362. 10.1007/s001250050832 9389430

[B19] Khwaja A. (2006). The role of Janus kinases in haemopoiesis and haematological malignancy. *Br. J. Haematol.* 134 366–384. 10.1111/j.1365-2141.2006.06206.x 16822289

[B20] Kurokawa T. Murashita K. (2009). Genomic characterization of multiple leptin genes and a leptin receptor gene in the Japanese medaka, *Oryzias latipes*. *Gen. Comp. Endocrinol.* 161 229–237. 10.1016/j.ygcen.2009.01.008 19523397

[B21] Leu J. Yan S. Lee T. Chou C. Chen S. Hwang P. (2000). Complete genomic organization and promoter analysis of the round-spotted pufferfish JAK1, JAK2, JAK3, and TYK2 genes. *DNA Cell Biol.* 19 431–446. 10.1089/10445490050085924 10945233

[B22] Li G. Liang X. Xie Q. Li G. Yu Y. Lai K. (2010). Gene structure, recombinant expression and functional characterization of grass carp leptin. *Gen. Comp. Endocrinol.* 166 117–127. 10.1016/j.ygcen.2009.10.009 19857495

[B23] Liu J. Shi Y. Gui J. (2005). Screening of differentially expressed genes at gastrula stage during embryogenesis of *Carassius auratus* gibelio. *Acta Hydrobiol. Sin.* 29 359–365.

[B24] Matarese G. Carrieri P. B. Montella S. De Rosa V. La Cava A. (2010). Leptin as a metabolic link to multiple sclerosis. *Nat. Rev. Neurol.* 6 455–461. 10.1038/nrneurol.2010.89 20606678

[B25] Murashita K. Uji S. Yamamoto T. Ronnestad I. Kurokawa T. (2008). Production of recombinant leptin and its effects on food intake in rainbow trout (*Oncorhynchus mykiss*). *Comp. Biochem. Physiol. B Biochem. Mol. Biol.* 150 377–384. 10.1016/j.cbpb.2008.04.007 18539064

[B26] Nassl A. M. Rubio-Aliaga I. Sailer M. Daniel H. (2011). The intestinal peptide transporter PEPT1 is involved in food intake regulation in mice fed a high-protein diet. *PLoS One* 6:e26407. 10.1371/journal.pone.0026407 22031831 PMC3198773

[B27] Nduati V. Yan Y. Dalmasso G. Driss A. Sitaraman S. Merlin D. (2007). Leptin transcriptionally enhances peptide transporter (hPepT1) expression and activity via the cAMP-response element-binding protein and Cdx2 transcription factors. *J. Biol. Chem.* 282 1359–1373. 10.1074/jbc.M604267200 16963449

[B28] Neubauer H. Huffstadt U. Müller M. Pfeffer K. (1997). Embryonic lethality in mice deficient in Janus kinase 2 (JAK2). *Immunol. Lett.* 57:275 10.1016/s0165-2478(97)86102-0

[B29] O’Shea J. J. Notarangelo L. D. Johnston J. A. Candotti F. (1997). Advances in the understanding of cytokine signal transduction: the role of Jaks and STATs in immunoregulation and the pathogenesis of immunodeficiency. *J. Clin. Immunol.* 17 431–447. 10.1023/a:1027388508570 9418183

[B30] Ostaszewska T. Kamaszewski M. Grochowski P. Dabrowski K. Verri T. Aksakal E. (2010). The effect of peptide absorption on PepT1 gene expression and digestive system hormones in rainbow trout (*Oncorhynchus mykiss*). *Comp. Biochem. Physiol. A Mol. Integr. Physiol.* 155 107–114. 10.1016/j.cbpa.2009.10.017 19854288

[B31] Pan Y. Wong E. A. Bloomquist J. R. Webb K. E. (1997). Poly(A)+ RNA from sheep omasal epithelium induces expression of a peptide transport protein(s) in *Xenopus laevis* oocytes. *J. Anim. Sci.* 75 3323–3330. 10.2527/1997.75123323x 9420008

[B32] Radosevic N. Winterstein D. Keller J. R. Neubauer H. Pfeffer K. Linnekin D. (2004). JAK2 contributes to the intrinsic capacity of primary hematopoietic cells to respond to stem cell factor. *Exp. Hematol.* 32 149–156. 10.1016/j.exphem.2003.11.006 15102475

[B33] Saltzman A. Stone M. Franks C. Searfoss G. Munro R. Jaye M. (1998). Cloning and characterization of human Jak-2 kinase: high mRNA expression in immune cells and muscle tissue. *Biochem. Biophys. Res. Commun.* 246 627–633. 10.1006/bbrc.1998.8685 9618263

[B34] Shimakura J. Terada T. Shimada Y. Katsura T. Inui K. (2006). The transcription factor Cdx2 regulates the intestine-specific expression of human peptide transporter 1 through functional interaction with Sp1. *Biochem. Pharmacol.* 71 1581–1588. 10.1016/j.bcp.2006.03.001 16616718

[B35] Sung J. Jeon J. Lee J. Kim C. (2009). Zebrafish Jak2a plays a crucial role in definitive hematopoiesis and blood vessel formation. *Biochem. Biophys. Res. Commun.* 378 629–633. 10.1016/j.bbrc.2008.11.116 19059211

[B36] Tartaglia L. A. (1997). The leptin receptor. *J. Biol. Chem.* 272 6093–6096. 10.1074/jbc.272.10.6093 9102398

[B37] Tartaglia L. A. Dembski M. Weng X. Deng N. Culpepper J. Devos R. (1995). Identification and expression cloning of a leptin receptor, OB-R. *Cell* 83 1263–1271. 10.1016/0092-8674(95)90151-5 8548812

[B38] Venkitachalam S. Chueh F. Yu C. (2012). Nuclear localization of lymphocyte-specific protein tyrosine kinase (Lck) and its role in regulating LIM domain only 2 (Lmo2) gene. *Biochem. Biophys. Res. Commun.* 417 1058–1062. 10.1016/j.bbrc.2011.12.095 22222369 PMC3264771

[B39] White M. F. (1996). The IRS-signalling system in insulin and cytokine action. *Philos. Trans. R. Soc. Lond. B Biol. Sci.* 351 181–189. 10.1098/rstb.1996.0015 8650265

